# Dancing in Your Head: An Interdisciplinary Review

**DOI:** 10.3389/fpsyg.2021.649121

**Published:** 2021-04-30

**Authors:** Andrea Zardi, Edoardo Giovanni Carlotti, Alessandro Pontremoli, Rosalba Morese

**Affiliations:** ^1^Department of Humanities, School of Human Sciences, University of Turin, Turin, Italy; ^2^Faculty of Communication, Culture and Society, Università della Svizzera Italiana, Lugano, Switzerland; ^3^Faculty of Biomedical Sciences, Institute of Public Health, Università della Svizzera Italiana, Lugano, Switzerland

**Keywords:** dance, mirror neurons, neuroaesthetics, motor imagery, embodied simulation, action observation, action understanding, motor expertise

## Abstract

The aim of this review is to highlight the most relevant contributions on dance in neuroscientific research. Neuroscience has analyzed the mirror system through neuroimaging techniques, testing its role in imitative learning, in the recognition of other people's emotions and especially in the understanding of the motor behavior of others. This review analyses the literature related to five general areas: (I) breakthrough studies on the mirror system, and subsequent studies on its involvement in the prediction, the execution, the control of movement, and in the process of “embodied simulation” within the intersubjective relationship; (II) research focused on investigating the neural networks in action observation, and the neural correlates of *motor expertise* highlighted by comparative studies on different dance styles; (III) studies dealing with the viewer's experience of dance according to specific dance repertoires, which revealed the relevance of choreographic choices for aesthetic appreciation; (IV) studies focused on dance as an aesthetic experience, where both the emotional and the cultural dimension play a significant role, and whose investigation paves the way to further progress both in empirical and in phenomenological research methodologies; (V) collaboration-based experiments, in which neuroscientists and choreographers developed expertise-related questions, especially focusing on the multiple phenomena that underlie motor imagery.

## Introduction

During the twentieth century, enormous progress has been made in the study of the central nervous system, as computational technology has evolved together with diagnostic tools to examine the structure and activity of the brain. Specifically, we refer to brain imaging or neuroimaging techniques: this term includes those methodologies that allow the functional investigation of the nervous system (Kandel et al., 2000). In fact, the above mentioned technologies have been used in experiments on the performing arts—dance in particular—in the wake of the discovery of mirror neurons. In the first section of this paper, the main steps in the investigation of the mirror mechanism are outlined, with particular attention to the cortical areas associated with the perception of movement and motor skills. This line of research aroused great interest also in the context of cultural studies on intersubjectivity, with contributions that found fertile ground in the phenomenology of Merleau-Ponty and Husserl (cfr. Gallese's approach to intersubjectivity and aesthetics).

The second section focuses on brain imaging studies (e.g., fMRI, TMS) in which the experimental task is the visual experience of dance. If, on the one hand, this line of research deals with strictly physiological mechanisms, especially those linked to the Action Observation Network (AON), on the other, a neurocognitive approach enables a wider investigation into the body-mind system and into the role of expertise in brain plasticity.

Sections Three, Four, and Five deal with several experiments which, building on seminal studies, widened the scope of the research. Here dance has been understood as a system of signs and actions ordered according to a specific technique and a choreographic score, which under specific conditions becomes an aesthetic and emotional experience. Some of these studies have pointed out the limits of the experimental setting and they have suggested hypotheses to recreate an ecological condition as similar as possible to live experience. Others have suggested a profitable coordination between empirical and phenomenological methodologies. Finally, others stressed the importance of analyzing ethno-cultural features and invariants. Generally, all of them emphasized the involvement of choreographers and professional dancers as an essential condition for designing and developing research projects.

There is no univocal approach in neuroscientific research on dance, as different methodologies have been implemented and multiple questions have been raised, often within the same experiment. In the attempt of defining the main topics of research, section Three of this paper presents studies where the viewer's experience is approached in the light of choreographic design, in order to determine which movements elicit significant responses, according to both empirical and phenomenological criteria. Section Four focuses on research related to the aesthetic and emotional features of those responses, often opening the way to new questions in neuroaesthetics and suggesting new methodologies to tackle its major issues. Finally, section Five reports experiments where dance professionals are the privileged subjects within studies focused on the relationship between expertise and motor imagery, of great importance for both choreographic and neurocognitive research.

### The Mirror System and Action Understanding

Recording the neuronal activity of macaque monkeys (Di Pellegrino et al., 1992), Giacomo Rizzolatti et al. discovered a mechanism based on “mirror neurons” (accordingly named Mirror Neuron System—MNS). It is a special class of sensori-motor neurons firing both at the execution and at the vision of an action, that was supposed to be the neural basis of the processes of imitation, understanding and prediction of other people's actions (also Gallese et al., 1996; Rizzolatti et al., 1996). By *understanding* it is meant “a bodily simulation of the behavior displayed by another person, but with the awareness that the behavior displayed is imbued with the mental life of the person displaying it: goals, intentions, but therefore also emotions.” (Caruana and Gallese, 2011, p. 231). Recording the neuronal activity of a macaque trained to recover objects from a box, when one of them performed an occasional gesture, the experimenters detected an anomalous activation in the animal's F5 area. The monkey's neurons were activated as if it had performed the action, while instead it had simply observed it (Di Pellegrino et al., 1992, p. 594). Discovering this peculiar propriety led to further research developments. Through the analysis of the role of neurons located in F5 area (Rizzolatti et al., 1988; Di Pellegrino et al., 1992), it was possible to differentiate which activations responded to the observation of a stimulus and which were related to the actual execution of movements.

Later, Rizzolatti and Fadiga (1998) highlighted the presence of two distinct classes of neurons: the so-called *canonical* ones and the *mirror* ones, with identical motor properties but slightly different visual properties. The latter reveal an activation that connects the observational act and the execution of an action. Through techniques such as positron emission tomography (PET) and functional magnetic resonance imaging (fMRI), it has been suggested that mirror neurons are present in humans (Fadiga et al., 1995) and that they have a role in imitation. This assumption opened the way to neurocognitive and neuropsychological studies both on the relationship between an action and the execution of an action and on the intersubjective relationship. Gallese (2001) suggests that mirror neurons are located in cortical regions provided with motor properties, because premotor neurons are able to establish a relation between expectations and results. The comprehension of an action could be described as an *embodied function*. The distinction between *canonical* and *mirror* neurons was also highlighted by Grèzes et al. (2003), underlining the activity of both types of neurons in the ventral limbs of the precentral sulcus.

In a subsequent literature review (Rizzolatti and Craighero, 2004, p. 179), an important correlation emerged between the observer's motor system and the observed action. An action that is not part of the subject's motor repertoire is represented only at a sensorial level, while the observation of an action which is part of the motor repertoire of the observer actually “resonates” within his/her motor system. By virtue of their visuo-motor properties, mirror neurons are able to coordinate the *visual information* with the observer's *motor knowledge*. Moreover, the activation of mirror neurons as motor neurons during the execution of an action not only codifies its “understanding” in terms of prediction or anticipation, but it is also linked to its execution in space and time (Iacoboni, 2005). Iacoboni et al. (2005) observed through fMRI that MNS is involved not only in the recognition of an action but also in the understanding the intentions of others.

Brodmann area 44, corresponding to F5 in monkeys, in the inferior parietal gyrus, has an important role in non-language related motor functions. Binkofski and Buccino (2004) summarized that a motor related part of Broca's area, known for the contribution to language production, is localized in Brodmann area 44. One of the cortical areas where mirror neuron-like activities seemed to be involved is precisely the Broca Area (Avikainen et al., 2001), where the production and processing of language takes place. This area has motor properties not strictly limited to verbal formulation. Its activation in coordination with the alleged mirror neuron regions hints to the possible development of language learning through different phases, starting from the recognition of mouth and hand movements (Buccino et al., 2001). Using rTMS (repetitive transcranial magnetic stimulation), a non-invasive technique that acts on predetermined areas by inhibiting their cortical activity, Heiser and his team have shown that the Broca Area plays a fundamental role in imitative processes (Heiser et al., 2003). The subjects of their experiment were required to simulate pressing a button with their finger when instructed by the graphics on a screen, while rTMS was activated on the anterior frontal convolution and on the occipital cortex. The inhibition of the process of imitating the requested action confirmed the essential role of the Broca premotor region in performing imitative actions related to fingers.

Research on the mirroring property of our brain has developed through the studies on human perception and on the aesthetic experience as a peculiar mode of perception. If, on the one hand, phenomenological studies, aimed at evaluating the role of the spectator, have always investigated the aesthetic experience within its usual environment, neurophysiological studies have had to deal with very binding environmental conditions and with evaluation parameters established by well-defined protocols and procedures. Yet, the discovery of mirror neurons marks a turning point in the studies on the perception of others, but above all on the human understanding of an aesthetic phenomenon based on the performance of actions. In fact, it highlights a physiological mechanism underlying our interpersonal relationships, which allows a direct understanding of other people's actions as a complex system of motor acts headed toward a final goal. These empirical findings can support and refine phenomenological research, insofar as the body is placed at the center of the investigation.

Through the body—in its manifestation of feelings, of intentions—the eminently relational nature of human behavior is realized: we “tune in” with others, with their emotions, their language and physical state (Gallese, 2010, p. 37). In particular: "The intentional tuning, generated by processes of embodied simulation, is consubstantial with the relationship of dynamic reciprocity that always takes place between the subjective and objective pole of the interpersonal relationship. [.] The study of the neural dimension of intersubjectivity and of the crucial role played in it by simulation mechanisms offer food for thought [.] for understanding on an empirical basis the active role of the viewer, and, more generally, for the mimetic origin of theatrical practices.”

The understanding of actions and intentions (Rizzolatti and Fabbri Destro, 2007; Canessa et al., 2012), the expression of emotions and sensations (Wicker et al., 2003; Bastiaanen et al., 2009), the development of linguistic skills (Mishra and Mohan, 2016), are all consequences of the intersubjective “resonance” for which the activities of brain areas with mirror properties are responsible (Vogeley, 2017, Gallese, 2003). Intersubjectivity is, with Vittorio Gallese's words, “a consubstantial dimension of our being humans” (Gallese, 2010). Similarly, Merleau-Ponty had pinpointed the role of corporeality in communication and intention: “The body is our general means of having a world. Sometimes it is restricted to the actions necessary for the conservation of life and accordingly it posits around us a biological world; at other times, elaborating upon these primary actions and moving from their literal to a figurative meaning, it manifest through them a core of new significance: this is true of motor habits such as dancing.” (Merleau-Ponty, 2002, p.194).

The studies cited so far offer an important contribution in this research field and are well-known studies on action observation. Recently (De Marco et al., 2020, p. 2) underlined how “observing motorically ‘familiar‘ actions relative to the own motor expertise determined a greater mirror motor activation (i.e., stronger motor resonance) and finer action prediction capability with respect to what happens while observing ‘unfamiliar‘ actions.” They considered other aspects beyond the classical conceptualization of the observation of the action, such as the kinematic characteristics of the movement (Hasson and Frith, 2016). As mirror neurons play an important role in the in understanding others' intentions (see for example Iacoboni, 2005; Gergely and Csibra, 2008; Ruggiero and Catmur, 2018) and also in clinical diseases (see for example Palermo et al., 2020). (Kilner et al., 2007, p. 161), they suggest that “the role of the mirror-neuron system in reading or recognizing the goals of observed actions can be understood within a predictive coding framework. Predictive coding is based on minimizing prediction error though recurrent or reciprocal interactions among levels of a cortical hierarchy.” In their perspective, they identify a precise role of mirror system in the ability to infer intentions from actions. Therefore, this recent evidence on the predictive coding framework, action anticipation, kinematic processing can contribute to a better understanding of the mechanisms involved in action perception not only from a neurophysiological perspective, but also from the point of view of dance studies.

## Perceiving Dance: Action Observation Network and Motor Expertise

Mirror neurons areas (inferior parietal and premotor cortices), however, can be envisioned as part of the Action Observation Network (AON), encompassing “all of the brain regions involved in action observation processes, rather than those exclusively engaged for observation and execution” (Cross et al., 2009), including areas related to visual analysis of action, and to visuomotor and sequence learning. It is therefore not surprising that experiments aimed at studying the perception of non-daily actions, and especially those of the performing arts (which started to be disseminated since 2005), focused on this broader network. As a preliminary remark, it should be noted that experimental protocols made use of video recording of dance movements, whereas performances take place live and the performers and the audience share the same space and time. However, video-recorded clips elicit significant brain activations, showing the occurrence of an experience that – though certainly different from the “original” —can shed light on the neural processing of performative acts. Moreover, live presence is not always mandatory for the spectator's emotional involvement and for the understanding of the complex neuronal process of perception (see also Jola and Reason, 2016).

In a seminal experiment, Calvo-Merino et al. (2005) presented classical ballet and *capoeira* short videoclips (4” each) to expert dancers in both styles and to non-dancers (control subjects) in the fMRI scanner. They detected a significant activation of the areas of the premotor cortex, in the intraparietal sulcus and in the upper lobe of the parietal area of ballet dancers at the view of ballet video and of *capoeira* dancers when the *capoeira* video was shown. Control subjects showed significantly lower activations in both cases. In expert dancers, activations matched the dance style of their expertise, showing that acquired motor skills possibly increase mirror activity. However, it is uncertain whether the result was due to visual habit and familiarity with the steps they watched.

A further experiment explored motor influences on action observation (Calvo-Merino et al., 2006) through observing gender-specific ballet moves. This research was carried out to assess whether visual or motor expertise prevailed on a gender basis. A set of gender-specific ballet moves video clips, in which the performer was alternatively a male and a female, and a set of video clips of ballet moves routinely performed by both genders, were shown to professional ballet dancers (males and females) during fMRI scanning. They had visual experience of all the steps, as that dance style is practiced in ensemble, though some of its steps are gender-specific. Data collected could therefore reveal whether the activation of mirror areas was more closely linked to a motor representation or to general processes of visual association: the brain's response to seeing an action depends on previous motor experience of performing that action, matching to the individual's motor repertoire. The activations of the premotor and parietal areas were found to be greater when the observer and the performer were gender-matched (e.g., a female observes the execution of a female step), thus suggesting a predominance of motor experience over visual experience.

In the same period, Cross et al. (2006) worked with professional dancers who were asked to learn and refine a choreographic sequence 5 h a week across 5 weeks. Once a week, they underwent fMRI and were shown videos both of the rehearsed sequence and of unpractised sequences as control movements. Afterwards, they had to evaluate their potential ability to perform the movements at best. During 5 weeks, as dancers' ratings constantly increased, the activity of the brain regions associated with both action simulation and action observation significantly raised, demonstrating that a complex motor resonance can be built in a short period of time. The premotor and parietal areas activity during action simulation was found to be enhanced by the acquired ability of execution, regardless of the dancers' familiarity with the stimulus or its semantics.

In a subsequent study, similarly based on matching dance training and dance observation, Cross et al. (2009) made the participants undergo fMRI scanning prior to and immediately after the days of training. Unlike the previous experiment, the volunteers were not dance professionals, and had to either simply watch or learn dance sequences with the help of a software connected with a dance-pad with sensors detecting foot position and timing. At fMRI scanning after training, parietal and premotor areas of the AON showed common activity for both trained and untrained, yet previously watched, sequences. Action resonance processes can therefore emerge in the brain even without physical learning, triggered by the mere observation of actions. This finding is especially interesting for its possible application in therapeutic contexts (which is beyond the scope of this review), the assessment of the role played by observational familiarity in dance appreciation, and its possible use in professional practice. These studies show that dance has become a privileged area of research, in so far as its steps can be isolated and tested as movement modules in order to analyze which neural networks underpin their observation, both by experts and non-experts. The results obtained have highlighted the role of expertise in increasing the extent of the response to the observation of practiced movements, so that more specific questions about the mechanism of embodied simulation were formulated (Gallese, 2019).

By the end of the first decade of the twenty first century, research proceeded by tackling more complex subjects than the neurophysiological correlates of dance observation. It expanded to the analysis of the spectator's experience of elaborate choreographic scores, as well as to the design of experiments involving dance professionals and their specific creative practice.

### Investigations on the Spectator Experience and the Dance Repertoire

Following these seminal studies, the aesthetic experience of dance and its neural basis has been investigated through research combining the technical knowledge of art with the analysis of the spectator's response to different stylistic patterns.

Calvo-Merino et al. (2008) developed their research focusing on the issue of dance reception. As stated by the authors themselves, this article can be considered the first neuroscientific study on the aesthetic perception in the performative context. It should be noted that previous neuroscientific investigation in the aesthetic domain had mainly focused on visual art (Zeki, 1999; Di Dio and Gallese, 2009; Kirk et al., 2009; Vessel et al., 2012) and on music (Koelsch, 2011). Within such experiments, the aim was to highlight the neural correlates of the aesthetic experience of dance: “brain areas whose activity during passive viewing of dance stimuli was related to later, independent aesthetic evaluation of the same stimuli” (Calvo-Merino et al., 2008). Inside the fMRI scanner, 24 short video clips (12 of classical dance and 12 of *capoeira*) were presented to non-expert participants, who were requested to perform an irrelevant task. In a second session they had to express a judgment for each clip using a five-point Likert scale, according to the following aesthetic dimensions: (1) simple–complex; (2) dull–interesting; (3) tense–relaxed; (4) weak–powerful and (5) like–dislike. After normalization, interesting results were found for the like-dislike (5) dimension, where high scores in subjective assessment corresponded to significant activations in both the visual and the premotor cortex, suggesting that a visual-sensorimotor network is recruited in the aesthetic experience of dance. Significant activations and higher scores were found to match in the case of jumps and whole-body displacements, while they were not found when the observed movements involved mainly one limb and minimal displacements, thus detecting a possible correlation between technically demanding movements and a greater aesthetic appreciation.

In the same years, however, other approaches to the aesthetic experience of dance were followed: in particular, Daprati et al. (2009) reflected on the reasons of changes in repertoire over time, especially focusing on the codified postures in classical ballet tradition.

The choice was not accidental, considering that between the eighteenth and the early twentieth century the academic dance code developed a fairly limited range of steps (dynamic/traveling movements like *glisser* or *tourner*) and postures (still positions like *relevé* Noverre, 1760), constantly influenced by the historical dialectics between “tradition” and “innovation” through the “archiving processes” of the body (Lepecki, 2010; Pontremoli, 2018). In addition, Rudolf Von Laban (Hutchinson, 2005) see Knust, 1959) cataloged and analyzed, through the study of choreology, the principles of movement according to a strict methodology. His notation system (*Labanotation*, see Benesh and Benesh, 1956) can serve experimental purposes by providing a rich database wherein dance movements are classified on the basis of four main components (body, effort, space, and shape), and associated to the expression of emotional states (Tschacor and Shafir, 2017).

Daprati et al. traced the evolution of a specific choreographic score over time, in parallel with changes in the audience's aesthetic taste. They focused on the prescriptive standards of dance, and started analyzing the variation in flexibility and strength of six different postures—from the “Rose Adagio” in different productions of *Sleeping Beauty* (Petipa/Cajkovskij)—, by measuring the elevation angle of the legs. Its constant increase across decades hinted to an evolution in dance technique, and accordingly to the progressive selection of professionals able to perform those variations on the academic vocabulary, that may be seen as a consequence of changes in aesthetic standards. In other words, the spectators' feedback seems to have affected the development of ballet aesthetics, demanding specific motor skills of the dancers.

In a second stage, the experimenters collected the aesthetic evaluation of dance-naive volunteers, who were requested to rate several blocks of geometric and stylized representations of the postures by indicating their preference. On average, they showed a preference for greater elevation of the lower limbs with respect to trunk inclination, hinting that extreme positions and movements seem to evoke a more intense primary sensory experience in the viewer.

The historical rising trend in physical standards, which affected the selection of the dancers, was allegedly a consequence of the constant feedback loop between the artists and their audience, and reflected upon the evolution of ballet aesthetics, extending beyond solo figures and major roles.

This study highlights a link between the development of dance aesthetics and audience feedback that would deserve to be investigated by extending the scope of the analysis to more similar samples, possibly with the aid of brain imaging techniques. In particular, it could be important to focus on the relevance of perceived difficulty on the aesthetic appreciation of dance movements and postures. Moreover, this line of research could benefit from testing experienced spectators of dance, using dance-naive participants as controls.

Later, Orgs et al. (2013) analyzed three levels of representation of the movement: body postures (symmetry and asymmetry), movement transitions and choreographic structure. A group of non-expert subjects (40) was dived in two subgroups: the first observed a set of symmetrical dance postures performed according to vertical and horizontal axes, the second observed asymmetric re-elaborations of the same postures. Finally, both had to rate the speed of the sequences and their aesthetic appreciation of them. In line with previous studies (Di Dio et al., 2007; Daprati et al., 2009), the participants' aesthetic evaluation was found to privilege spatial geometry, as symmetrical body positions, both vertically and horizontally, were preferred. As far as movement transitions are concerned, the level of aesthetic appreciation is greater when the sequences appear simple and fluent. Overall, this study provides choreographic designers with useful hints about how spatial relationships among bodies and movement transitions can affect the aesthetic appreciation of dance.

The availability of brain imaging techniques suggested instead to investigate the possible relationship between the aesthetic appreciation of a movement and AON activations. Cross et al. (2011) posited that the observer's appreciation is linked to his/her perceived ability to reproduce the movement observed.

Twenty two participants, all with limited or no dance experience, were asked to evaluate their ability to replicate the movements performed on video by professional ballet dancers, while they underwent fMRI scanning. A significant activation of the AON (Grèzes and Decety, 2001; Cross et al., 2009; Gazzola and Keysers, 2009) in the occipital and part of the parietal area; on their side, participants reported to prefer movements they perceived to be beyond their performing ability, and declared to appreciate the fluidity of the dancers' execution. A significant correspondent activation within the IPL (Inferior Parietal Lobule) suggested the possibility that the observed movement outside the performing ability were subject to an embodied simulation process, related to the visual-spatial ability of this area. In fact, as the neurons of the IPL integrate visual, auditory and somatosensory stimuli for action and perception of the external world, it has been suggested that they are the basis for the awareness of the body in space, in association with the SPL (Superior Parietal Lobule), that mostly processes proprioceptive inputs (Gallese, 2007a,b). It also emerged that EBA (Extrastriate Body Area, a posterior portion of the middle temporal gyrus, near the inferior temporal sulcus, that responds to static representations of the human body or still body parts; see Cross et al., 2010) activations were increasing with the viewer's perception of the gap (in flexibility, strength, athleticism) between her/his body and the performer's one. Aesthetic appreciation and perceived motor difficulty are therefore strongly linked in the observer's experience, and distinguished by peculiar activations of the AON.

In 2011, Corinne Jola et al. focused again on ballet repertoire, trying to create an experimental setting for an entire performance, using the dress rehearsal of *Sleeping Beauty* at the Theater Royal in Glasgow. Their unusual research mainly aimed at demonstrating that environmental and ecological conditions radically influence results, drawing attention to the limits of the laboratory environment. Secondly, the experiment aimed at analyzing the effects of a prolonged experience of a performance on the cortical area. Due to obvious practical reasons, transcranial magnetic stimulation (TMS), associated with electromyography (EMG), was used to stimulate and verify the spectators' motor cortical excitability response. In this setting, the results showed a decrease in the cortical response in time, referable to a progressive adaptation to the procedure and the consequent effect of muscle relaxation. The authors found significant variations in both time and individual responses, though the limited number of participants (4) does not allow to draw definite conclusions. Moreover, as they attended at a performance structured in a narrative form, the attention. It should also be considered how that condition could impact on the results, as the narrative progress can possibly turn out to be a major cause of inhomogeneity in the observer's attention during the whole session. Notwithstanding all its inherent limitations, in any case this study for the first time, attempted to highlight that empiric research on dance needs to take into account protocols and settings according to ecological criteria.

Later, Jola et al. (2012) chose again live performances in a study that will be of great importance for subsequent research on this topic: by using TMS, they wanted to test the impact of the visual experience of dance on motor simulation, by measuring corticospinal excitation (MEPs, Motor Evoked Potentials: see Carson et al., 2016). Thirty two volunteers—without any practical dance experience—watched three small 5 min live pieces: ballet, Indian dance (*Bharatanatyam* dance) and a silent piece performed by an actress. This last piece served as a non-danced control condition, designed to match the two danced pieces with regard to the use of space and body actions (clapping hands, stomping feet, showing fists). Spectators and performers shared the dance hall at the same time. The participants were selected to form three groups: experienced spectators of ballet (1) and of Indian dance (2), and “novices” without spectatorship experience (3). The groups of experts displayed a corticospinal excitability corresponding to the related muscle activity of the arms and fingers of the performers: a sort of participatory “mirroring” with their own muscularity. Indian dance expert spectators also showed high scores on the fantasy subscale of the Interpersonal Reactivity Index (IRI; Davis, 1980; Keaton, 2017). Although some choices in the experimental condition could be refined, as the authors duly highlight, the results show that not only motor expertise, but also visual experience is able to modulate the response to the observation of movements, by eliciting a covert internal simulation of the movements observed, in line with previous findings (Cross et al., 2009). Moreover, IRI results analysis, in correlation with MEPs, points out a relevant role of empathic abilities in this internal simulation. In summary, the experiment opens the way to approach a series of questions concerning visual familiarity and emotional involvement, which subsequent studies on the aesthetic experience of dance have endeavored to tackle.

### Aesthetic Appreciation and Emotional Experience of Non-Dancers Spectators

Accordingly, a further line of research has been developed to study the neural correlations between the visual experience of dance and emotions. In this line, the aesthetic experience is approached as an emotional response which has to be investigated according to broader perspectives, including cultural biases and phenomenological assessments.

In order to tackle the emotional response, Grosbras et al. (2013) first scanned through fMRI 16 participants without dance experience, showing them a 3'33” video of contemporary dance (*Double Points: 3X* (http://tinyurl.com/bol4nsg), a duo by Rosie Kay, characterized by a strong dynamic and emotional intensity) with three different soundscapes: the rhythm of the breath of the dancers, techno music and classical music. Subsequently, the same participants were asked to rewatch the video outside the scanner and to rate the intensity of their emotions as spectators through a cursor. fMRI analysis revealed a negative correlation between the emotional judgment and the activation of the right posterior parietal cortex. In a further session, a second group of 18 subjects without dance experience watched the same video, while they underwent rTMS on the above mentioned cortical regions, to verify if changes in their subsequent emotional evaluation of performance were related to an increase in frequency (positive evaluation). The results confirmed the negative correlation between the ratings of emotional reaction to dance and the activity in the posterior parietal cortex, an area associated more with cognition than emotion (Drevet and Raichle, 1998). The inhibition of the parieto-frontal control system through TMS reduced the cognitive processing of the dance and enhanced the emotional response. The analysis of the questionnaires (Likert Scale), where the participants pointed out the moments of greater emotion, showed that the perception of fluid movements well-synchronized to music was instrumental for increasing their affective reaction.

This research was subsequently developed by Reason et al. (2016), who focused on the analysis of the visual experience of the same dance piece with different soundscapes: a Bach *Concerto*, the dancers' breathing and footfalls, and electronic music, positing the importance of the auditory stimulus for the spectator's experience. The team followed an elaborate procedure: in the first stage a group of participants watched the piece in a theater; after the performance, they were divided in two sub-groups and they underwent a session of phenomenological data collection, that highlighted a significant experiential difference between the piece with Bach's music and the piece with the sounds generated by the dancers, whereas the electronic music soundscape did not stimulate notable subjective responses. Subsequently, those two versions were used as video recordings to test 22 other participants (who had not watched the live pieces) through fMRI scanning. Results were analyzed using a data driven technique, ISC (Intersubject Correlation, Nastase et al., 2019), in order to highlight the common processes in brain regions across the group. As expected, when the video had the soundscape of Bach's *Concerto* the fMRI scanning was played with the superior temporal gyrus, involved in processing complex sounds, while activations in the parietal cortex (ventral and dorsal of Brodmann area 6), implicated in the motor cognition and aesthetic processing of dance, were found under both audio conditions. In the case of the environmental soundscape (breathing and footfalls), an activation of the posterior region of the same area was found, suggesting a greater involvement of the body—a *kinaesthetic empathy*: in the words of Hagendoorn, “when watching dance the observer is in a sense virtually dancing along” (Hagendoorn, 2004). However, despite the common body response across the group, there was a significant difference in the subjective aesthetic and emotional evaluations expressed by the participants, pointing out more questions than conclusions. This research shift neuroscientific method of recording toward a closer approach to the ecological context of performance enjoyment. Analyzing the results in a general view it is possible to understand how this approach builds bridges between the fields of motor physiology and aesthetic perception studies. Exploring the relationships with emotion and perception on the one hand and sound/sight interaction on the other, it is possible to open up new questions related to choreographic creation and the use of sound and visual instruments within the performance.

The role of the co-presence of sound and movement in the aesthetic perception of dance—contemporary, in this case—has also been analyzed by Howlin et al. (2018). Thirty four participants (more than a half with previous dance education) viewed a recorded version of *Group Study*, choreography by Matthias Sperling (33'54”). Their quantitative aesthetic response was measured with the CAM Examination Model (Congruence-Association Model), while their qualitative response was collected through a questionnaire where they had to comment on several categories (movement/sound/synchrony/boring/unpleasant/search for meaning/enjoyable). The dance performance did not include any music, except for the breathing, the counting, the humming sounds of the dancers, and it was presented to three sub-groups of volunteers, who watched the recording (1) with its congruent soundscape; (2) with the soundscape played in reverse (incongruent); (3) without any soundscape.

The subjects reported a greater enjoyment of the performance when there was an inconsistency between sounds and movement: that is, when a sound was “dissociated” from its original movement, the spectator had a greater involvement. The occurrence of a kinaesthetic empathy between the performer and its audience as a trigger for positive aesthetic judgements is thus questioned, whereas the absence of congruence between movements and sounds seems to produce in the viewer an increase of attention, produced by his/her expectation of possible synchronies.

As it has been already noted, the experimental limitations of brain imaging technologies prevent the reproduction of the spectator's usual condition, which is strongly affected by both the live presence of the performer on stage and the feedback mechanism involving and connecting the performer and the audience. This condition implies that the experience cannot be approached without attempting to reproduce, at least in part, the relationship between the artist and the audience and the intersubjective mechanisms that allow the viewer to enjoy the performance (Sofia, 2013) in an ecological context.

The aesthetic experience of dance, in terms of a condition influenced by the live presence of the performer and of an audience, was analyzed by Jola and Grosbras (2013). They developed the procedure of a previous experiment (cf. Jola et al., 2012, above) to tackle the issue of its ecological validity by comparing the motor resonance of two groups of non-expert subjects while watching three 5 min solos (classical ballet, an Indian *Bharatanatyam* dance, and an acting control condition), one group in video and the other live. The primary motor cortex was stimulated through a single-pulse TMS under different viewing conditions, while electromyography (EMG, Abbruzzese and Trompetto, 2002) recorded the increase in neuronal excitability of corticospinal motor neurons (MCE), indicated by larger Motor Evoked Potentials (MEPs) amplitudes in corticospinal tract (Petersen et al., 2003; Legatt, 2014). The experimenters detected a higher MCE increase in the muscles of the arm of those who watched the three live performances, varying through the duration of each solos, in comparison to the group that watched the video (*Bharatanatyam* dance was the performance that activated MCE the most, being richer in gestural elements). Moreover, the subjects of the “live group” reported in psychometric questionnaires (Likert scale) a higher enjoyment score than the “video group.” The merit of this study was to collect data on the difference between live and technologically mediated experience of dance. Its results highlight an issue that, during the closure of theaters because of the present pandemic crisis, is still more noteworthy.

Researchers have constantly attempted to implement the framework of knowledge, which can be used to approach the aesthetic process that underlies the spectator's experience of dance. In 2013, Christensen and Calvo-Merino reviewed the literature hitherto published, focusing on the connection between emotions and aesthetic experience. According to them, a new perspective on empirical aesthetics can be opened by concurrently considering the involvement of the neural networks related to the representation of the body (Calvo-Merino et al., 2008), the aesthetic perception (Cross et al., 2011), and the emotional response (Grosbras et al., 2013). Furthermore, Christensen and Calvo-Merino suggest that the study of some dance positions, cross-cultural and part of different repertoires (e.g., *attitudes*, à* la seconde, cambrées*), has to be developed, in order to assess the importance of anthropological and ethnic-cultural dance invariants. Accordingly, they invite to consider the communicational and social functions of the aesthetic experience of dance, without isolating a single movement from its choreographic context that generates much of its emotional meaning.

Cross and Elizarova (2014) also brought attention on the need to extend the scope of the research on dance by reviewing almost 10 years of literature and remarking that the neuroscientific advancements in action and perception studies, which have exploited the collaboration with dance professionals, were relevant on both sides. Accordingly, the authors suggest designing future experimental protocols by implementing new forms of collaboration, according to possible respective advantages. For example, choreographers may benefit of research involving the performer-spectator relationship; scientists may find new ways to investigate human behavior. Actually, those suggestions have been followed in several subsequent studies, as we will presently see.

Christensen et al. (2017) published another review containing several perspectives to depict the importance of dance for the human condition; dance is defined as a behavior dating back to the beginning of the humankind. The reviewers indicate six functions of dance, all connected to neurophysiological mechanisms, to be considered and used to broaden the scope of research.

1. Dance induces a state of flow (Csikszentmihalyi, 2008): creative activities re-establish biochemical balances in order to attain a state of happiness and a sense of purpose. Ulrich et al. (2013) demonstrated through fMRI that the flow influences neural activity in brain regions (IFG, AMY, MPFC), bringing a deeper sense of cognitive control and then a decreased negative arousal.
2. Basic emotional experience: mesolimbic dopaminergic–amygdala systems control changes in relaxation and stress relief, activating parasympathetic nervous system activation and provoking the biological need to dance or to watch dance.
3. Imagery: Dance provokes an intense activity of imagining and performing: dancers use imagery in their practice and non-dancers also experience imagery episodes from watching a dance.
4. Communication: the comprehension of emotions and intentions expressed through dance is related to resonance mechanisms through which we understand everyday actions and this mechanism is enhanced by training.
5. Self-intimation: the way a body is placed in space, the alignment of the limbs, muscle contractions in large muscles, and the contractions of the facial muscles are the genuine expression of a person's inner state.
6. Social cohesion: dancing together involves processes of affection. Moving together stimulates the limbic brain, which controls the release of oxytocin, and homeostatic agents.

In a previous review, Kirsch et al. (2016) also focused on the complexity of the aesthetic experience, and approached the topic by highlighting the activation of a network of cerebral areas involving reward and motor processing. In particular, studies concerning the aesthetic perception of the human body in visual and performing arts were analyzed in relation to the training of the experimental subjects. Both perceptual and motor training have been found to be effective in modulating the aesthetic experience of dance, allowing the appreciation of specific performance components (e.g., grace, strength, precision of movements) (Montero, 2011; Kirsch et al., 2015). Moreover, fMRI scanning ante- and post-training showed a significant shifting of brain activations from subcortical regions associated with the dopaminergic reward system to posterior temporal regions recruited in processing multisensory integration, emotion, and motion (Jola et al., 2013).

These processes presumably contribute to the plasticity and ductility of neural mechanisms involving the aesthetic experience. The individual character of the aesthetic experience raises further questions to be duly explored.

The correlation between brain activations and aesthetic appreciation of dance has mainly been analyzed in experimental settings, though several studies called attention to the relevance of ecological protocols, maintaining that an investigation on the empirical aesthetic of dance has to consider its status as an ephemeral art, whose sensory stimuli are not as stable and continuous in time as those of the visual arts (Christensen and Jola, 2015). Moreover, the importance of accounting for auditory stimuli as well-encouraged empirical research on the spectator's aesthetic experience (Christensen and Calvo-Merino, 2013; Jola et al., 2013; Christensen and Jola, 2015) to take advantage from the studies on performance as a multisensory experience (Banes and Lepecki, 2007; Di Benedetto, 2010; Blasing, 2015), where visual and auditory stimuli are jointly analyzed as a distinctive feature of the experience. In support of this approach, a recent study by Pollick et al. (2018) demonstrated how the regions of ISC increase in performances with audio, revealing the network organization of regions sensitive to audiovisual presentation (ISC maps also show an increase when audio and video editing effects are added).

In his theoretical analysis of previous literature on the basis of linguistic communication framework, Orgs et al. (2016) defines the aesthetic impact of dance as “a result of successful message-passing between performer and spectator” (p. 2). Starting by postulating bidirectional and interactive communication, Grice's “cooperative principles for successful conversation” are used to describe two communication stages: the syntax of postures/movements/sequences, and their semantic universe (Grice, 1975). As previous studies confirmed that the aesthetic appreciation of movement strongly depends on the spectator's motor expertise (Bläsing et al., 2012; Jola et al., 2012, 2013), the correlation between the familiarity with the movement and the complexity of information was used to evaluate the generation of aesthetic responses as not exclusively linked either to the message, according to an objectivist perspective, or to the viewer, from a subjectivist perspective. The aesthetic processing was analyzed as a two-way, interactive communication, where the individual appreciation relies on personal aesthetic preferences and acquired experience. In summary, the aesthetic judgment is related to the relationship between the clarity/simplicity/familiarity of the message and the mastery of the language used (Noble et al., 2014).

More recently, Orlandi et al. (2020b) approached the aesthetic experience of dance analyzing how movement kinematics can affect the observers' evaluation. They presented their experimental subjects (non-experts) with video clips of a dancer performing the same sequences (played forward and backward) with timing variations. During two different sessions, participants were asked firstly to evaluate the perceived speed of each movement and the effort required to its execution, secondly their enjoyment of the sequence and their ability to reproduce it. Each sequence underwent a quantitative assessment according to speed, acceleration, motion energy, motion smoothness, and motion entropy. Results showed that slowness and uniformity of the movements are less appreciated than fastness and variableness, which are perceived as signs of effortfulness, though slower and uniform sequences are actually more demanding to the performers. The novelty point of the research is that the effective communication in dance is linked to kinematic variability and predictability and directly modulates the aesthetic pleasure associated with dance observation. Moreover, this study confirms that the aesthetic evaluation of movements is linked to their perceived difficulty, as previous experiments pointed out; yet, in addition, it highlights that movement timing is a significant parameter for appreciation, and its investigation across cultures and styles can contribute to dance aesthetics.

The above-mentioned studies reveal that the spectator's aesthetic experience of a dance performance is conceivable as a multi-dimensional phenomenon, connected to physical and biological aspects like timing, shapes, choreography, effortfulness, setting condition, which contribute to the observers' satisfaction and sense of fulfillment. The assessment of these aspects may help dancers and choreographers to redefine the creative process according to the potential reception on the part of the spectator.

### Motor Imagery: an Interdisciplinary Approach

The studies hitherto reviewed depict the passage from a first stage, when dance served as a fertile ground to understand the mechanisms underlying action and perception, and the relevance of mirror activity, to a second stage when it was the basis for an empirical approach to aesthetics, later enriched with phenomenological and ecological concerns. As dance co-evolved with the human being, it “has maintained a critical presence across all human cultures, defying barriers of class, race and status” (Cross and Ticini, 2012, p. 5), the focus of research moved toward the spectator's experience and its neural correlates.

A certain number of studies have dealt not only with the neural processes in the spectators, but also with the multiple neurocognitive phenomena that occur in dancers during the performance. Bläsing et al. (2012) comment on the research that has been carried out in this area highlighting significant aspects. First, technical training increases sensorimotor mastery and proprioceptive skills (Golomer and Dupui, 2000; Jola et al., 2011) and dancers perform balance and position-matching tasks by relying more on proprioception than visual ability in dynamic position tasks. In contrast, a greater level of error rates, in a position-matching task, has been found when they move in sensorimotor dominance of a visual condition. In fact, during a balance task, dancers rely more on visual information rather than proprioception: empirical evidence showed that dancers with closed eyes perform no better than controls.

Second, Bläsing et al. reviewed studies about timing and synchronization (Vicary et al., 2017) in complex collective choreography (Maduell and Wing, 2007), identifying several variables that affect the performance of choreographic *ensembles*:

- *Attention*: complex choreographic patterns and tight spaces increase the margin of error in timing (Mivielle-Moncla et al., 2008). Synchronization with other dancers requires more attention to the time factor.
- *Rhythm*: the use of musical cues not only helps in observing choreographic timing, but it is also a support. Physical contact between dancers facilitates timing with the metric structure of music (Gentry and Feron, 2004; Toivianen et al., 2010).
- *Motor experience* of the performer increases not only the synchronization of familiar positions, but also of unfamiliar ones, as well as the ability to make up for the errors of other dancers (Honisch et al., 2009).

Moreover, dance practice increases the imaginative capacity of the kinaesthetic sensation, facilitating the body's interaction with the surrounding space and coordination and increasing visual motor skill and spatial adaptation (Golomer et al., 2007). Practice also increases the tool to learn and optimize movements, to create new material and to exercise the memory of long choreographic sequences (Fink et al., 2009; May et al., 2011). As Jeannerod and Frak (1999) have observed, motor imagery is related to a cortical/subcortical network sharing several areas with motor execution (premotor cortex and cortical areas involved in motor execution, lateral cerebellum, basal ganglia) and action planning (dorsolateral prefrontal, posterior parietal and inferior frontal). Overall, this line of research intend to find the connection between the performer's physical and dynamic experience and the perceptual and aesthetic experience of the viewer (as anticipated by Arnheim, 1974). It is necessary to underline how the choreographer's aesthetic approach to composition can benefit from the experimental outcomes, and choreographic research can be channeled, enriched, and “validated” by the interdisciplinary dialogue (Jola, 2010).

Dance, as a performing art, implies the involvement of a spectator and it is precisely this relationship that has been investigated both from a phenomenological and a sociological point of view, and within neurocognitive studies. In particular, several research carried out through fMRI or TMS have highlighted the importance of a sensorimotor mechanism to evaluate the aesthetic experience of dance, a network of activations that measures the appreciation of dancing body postures. The relationship between the enjoyment of the vision of dance and the lack of physical ability appears to be mediated, as already seen (Cross et al., 2006), by parietal and occipital cortices, reflecting a high level of embodiment during the perception of dance.

May et al. (2011) focused on the mental strategies that underlie contemporary choreographic research. The authors designed a two-phase experiment to investigate how the ability of imagining a movement is involved in its creation, based on the technique practiced by choreographer Wayne McGregor and by Random Dance dancers (https://waynemcgregor.com). In the first experiment, choreographic exercises were followed by self-assessment forms: the EIS (Schutte et al., 1998), a *self-report* aimed at acquiring a characterization of the dancers' creative experience and reflection about their beliefs, and the *Thought Monitoring Exercise*, in order to precisely understand the image in the subject's mind at a specific time. Subsequently, the dancers were asked to write choreographic scores only mentally—i.e., in a “static” condition—and in a conventionally dynamic condition: the static condition was analyzed through fMRI, which highlighted activations in the areas of the orbitofrontal cortex connected to assessment and decision-making skills (Kringelbach, 2005), of the mid-temporal region, related to the representation of the human body and dynamic action (Downing et al., 2001, 2006), and of the occipital cortex. The lateral occipital cortex is a region of the human ventral stream known to be involved in the visual recognition of objects, in particular of their geometric structure (Goodale, 2011). This exploratory study indicates that the neural and experiential qualities of imagination during the creation of movement can be subject to scientific investigation. Its results are of great interest also within the artistic ground. For instance, this new approach to the creation of dance movements increased the ability of the dancers to create new choreographic strategies. In fact, the fMRI data analysis showed that the practical knowledge of space (spatial-praxis) and the imaginative visualization activity share activations in the orbitofrontal cortex, linked to the decision-making ability and to the creation of expectations, with additional activations in the temporal region (representation of the human body, see Downing et al., 2006) and the occipital cortex. Actually, this research contributed to the development of the Choreographic Thinking Tools project (deLahunta et al., 2012; Barnard and deLahunta, 2017) and to interesting and fruitful artistic results.

The birth of several projects aimed at networking artists and neuroscientists in a common research is seen as an example of this cross-disciplinary perspective (Cross and Elizarova, 2014). One of the most relevant is Motionbank (www.motionbank.org, deLaHunta, 2016; Gioia Monda, 2016; deLahunta et al., 2017): a 4 year project by William Forsythe, aimed at creating digital dance scores and building relationships between dancers and researchers in different disciplines, under the Dance Engaging Science Group (Vass-Rhee, 2013). This is an example of interdisciplinary research taking place in the liminal territory between artistic practice and scientific investigations, where the choreographic creation is influenced by the results of experimental research and in its turn can suggest new lines of investigation to future studies.

Experiments aimed at analyzing the neural correlates of motor imagery in dancers also contributed to deepen the understanding of certain brain areas. For instance, the role of the EBA (Extrastriate body area) and the FBA (Fusiform body area) in the occipitotemporal cortex (OTC), involved in the perception of the body of others and its parts, was identified by studies carried out through fMRI (Downing et al., 2001) extended to TMS (Downing and Peleen, 2011). A recent research by Orlandi and Mado Proverbio, 2019 investigates the effects of professional dance training in modulating the functional response of the OTC at an early stage of the processing action. Expert ballet dancers (17) and non-dancer controls (17) were shown 326 short videos (2 s) depicting a series of technical movements belonging to the ballet repertoire. They were instructed to observe each video carefully and subsequently simulate the movement mentally, during EEG recording. The observation of dance movements elicited a larger occipitotemporal negative component (N2) over the left hemisphere in dancers compared with controls. The swLORETA (standardized weighted low-resolution electromagnetic tomography) source reconstruction revealed the engagement of bilateral inferior and middle temporal regions (together with visuomotor cortices) in dancers, and right-lateralized activity in controls. In addition, the dancers (relative to controls) showed faster movement processing and enhanced recognition, as indicated by the early P2 and enhanced P300 responses. These pieces of evidence suggested increased functional symmetry of the early OTC (Occipitotemporal Cortex) response to dance kinematics, due to long-term whole-body practice.

Another recent research (Paris-Alemany et al., 2019) demonstrates how motor expertise increases motor imagery skills in dancers, highlighting two typologies: visual motor imagery as a process of self-imagination in the execution of a motor task and kinaesthetic motor imagery, which consists in the somatosensory execution of a motor task (Fery, 2003). Both these processes share the same neuronal networks (Filgueiras et al., 2018). Paris-Alemany et al., carried out a cross-sectional study analyzing the differences in the imagination processes between 15 flamenco dancers, 15 classical dancers and 15 contemporary dancers. They were asked to imagine a specific arm extension movement (task 1) and a jump (task 2) and to evaluate the difficulty of vision/imagination through a scale from 1 (very difficult) to 7 (very easy) through the MIQ-R Questionnaire (Hall and Martin, 1997). Responsiveness has been timed. The results demonstrate a predominant visual mode to imagine jumping in flamenco dancers, because it is unfamiliar with their motor repertoire. The time taken to perform the movement and to generate mental images is related to the familiarity with the movement itself: performing an unfamiliar movement involves more time in viewing the movement and in the execution.

The kinaesthetic motor imagery was also analyzed by Orlandi et al. (2020a). In motor imagery, the frontal and parietal motor areas have been proved to be involved (Fleming et al., 2009): their research aims at investigating the neural correlates of imagining a complex action and how it affects the expertise. Sixteen professional and ex-professional classical dancers and 16 university students were involved as control subjects. Tested via EEG, they were asked to mentally reproduce ballet movements previously watched in video. These were effortful movements (*sissonne sauté, arabesque penchée, grand jeté à la seconde*), and some effortless movements (*changement, tendu croisé*). The source reconstruction of the potentials recorded through the EEG (AN, anterior negativity) showed an increase in the activation of the prefrontal regions (Brodmann Areas 10/47) in the control subjects, while the occipitotemporal (BA 20) and bilateral sensorimotor areas were more significantly active in dancers. This result highlights that kinaesthetic motor imagery uses a sensorimotor visualization process in subjects with acquired dance experience, while greater cognitive effort is required for untrained subjects.

## Conclusion

Neuroscientific studies on dance have hitherto provided significant evidence on the role of sensorimotor mechanisms in the execution and the observation of movements. They added new elements to the notions of “internal resonance” and “embodied simulation.” Concurrently, from the perspective of neuroaesthetics, the technical, environmental, and cultural conditions determining the spectator's experience of the performing arts raise more questions than answers. These cannot be tackled without coordinating the empirical investigation with a reliable phenomenological approach: yet, the present technological limitations prevent the collection of potentially valuable data and more refined first-person report methods are yet to be implemented. Nevertheless, research progress highlights that dance can be a fertile field of study also from a reductionist point of view, as the analysis of the neural correlates of actions and movements has shed light on broader topics concerning human behavior.

In future perspective, the above-mentioned lines of research can be further developed through the contribution of dance professionals in drafting hypotheses and the implementation of experimental protocols (in this regard, a significant step has been taken by Christensen et al., 2019). Accordingly, it is necessary to stimulate interdisciplinary dialogue, in order to design new projects aimed at collecting data with a greater attention to ecological settings and conditions. At the same time it would provide information on the stylistic and cultural variables that characterize different dance genres in different contexts. In developing this perspective, it would be also desirable to confront the issue of inductively defining what dance is, by paying attention at which genres (e.g., codified or free-from) elicit significant responses in the observers, both empirically and phenomenologically, under different contextual conditions.

Finally, it is important to underline that, in the last few years, the creative processes in choreography themselves recognized the value of the neuroscientific approach, which stimulated a broader reflection on the perception of dance within specific artistic and cultural contexts. In particular, the current health emergency, strongly limiting live performances, has accelerated—with different outcomes—the creation of choreographic devices exploiting the execution of performance modules not in presence: the artists' attention to neuroscientific research methodologies has fostered their awareness of the use of technological tools and its implications on audience reception.

## Author Contributions

AZ conceived the content of the article and wrote the manuscript. EC contributed with the technical revision on the performance studies. AP and RM reviewed the manuscript. RM in the role of senior and principal investigator supervised the paper applying an interdisciplinary approach. All authors contributed to the article and approved the submitted version.

## Conflict of Interest

The authors declare that the research was conducted in the absence of any commercial or financial relationships that could be construed as a potential conflict of interest.
